# The roles of residential greenness in the association between air pollution and health: a systematic review

**DOI:** 10.1088/1748-9326/ac0e61

**Published:** 2021-08-24

**Authors:** Ji-Young Son, Hayon Michelle Choi, Kelvin C Fong, Seulkee Heo, Chris C Lim, Michelle L Bell

**Affiliations:** School of the Environment, Yale University, New Haven, CT, United States of America

**Keywords:** air pollution, effect modifier, health, residential greenness

## Abstract

While a growing body of literature suggests beneficial impacts of greenness on several health outcomes, relatively few studies have examined greenness as an effect modifier to impacts of air pollution on health outcomes, and results from the existing studies are inconclusive. We performed a comprehensive, systematic review of previous literature on greenness as a potential effect modifier for associations between particulate matter air pollution and health. After initial screening of 7814 studies, we identified 20 eligible studies. We summarized findings on study characteristics based on several criteria: health outcome, air pollution exposure, source of air pollution data, study location, study period, and median year of the study period. We evaluated characteristics of effect modification by greenness on air pollution and health associations based on the number of greenness metrics applied, type of greenness metric (e.g. normalized difference vegetation index, land use), data source for greenness, and spatial resolution and buffer size. We also summarized evidence for effect modification by greenness based on strength and direction of evidence for each study and overall evidence of effect modification by greenness by several study characteristics. Our systematic review showed that only a limited number of studies have been conducted on greenness as an effect modifier for air pollution-health associations. We found differences in several study characteristics such as greenness assessment (e.g. greenness metrics applied, spatial resolution, and data sources) across studies. Collectively, the studies provide suggestive evidence for the hypothesis that areas with high greenness have lower impacts of air pollution on health, although some studies reported inconsistent findings. The findings from our review provide valuable knowledge on how greenness affects associations between air pollution and health and could help identify critical areas for future study.

## 1. Introduction

More than half of the world’s population currently lives in urban areas and the world’s urban population is expected to increase to 68% by 2050 (United Nations 2018). Urbanization can bring a number of benefits such as economic growth and development, but also adverse impacts such as urban sprawl, pollution, and environmental degradation. Greenness is known to have environmental benefits such as reduced air pollution, noise, temperature and urban heat island effect by filtering of air pollutants and increased deposition and dispersion; absorption, refraction, deflection and masking of sounds; and decreased local temperature due to shade and vegetation (Janhäll 2015, Van Renterghem et al 2015, Aram et al 2019). Many previous studies investigated the direct effect of greenness on health and suggested that exposure to green spaces is associated with better health outcomes such as reduced mortality and improved mental health (Gascon et al 2016, 2018). However, in addition to the direct impacts on health, green space appears to modify the association between other environmental conditions and health outcomes. The mechanisms of the modifying effects of greenness on the associations between air pollution and health include general health enhancement through increased physical activities, stress reduction, changes in the chemical composition and mixture of pollutants, more social interaction, and other factors impacting health in ways that affect susceptibility (Sæbø et al 2012, Markevych et al 2017, Franchini and Mannucci 2018).

While a growing body of literature has suggested beneficial impacts of greenness on several health outcomes such as mortality, cardiovascular outcomes, mental health, physical activity, asthma and allergy-related symptoms, and birth outcomes (Banay et al 2017, Fong et al 2018, Zhan et al 2020, Hartley et al 2021), fewer studies focused on greenness as a potential effect modifier to the impacts of air pollution on health. Recent studies suggested lower impacts of air pollution on health for those living in areas with high greenness and access to green spaces, including studies on mortality and preterm births (Asta et al 2019, Kim et al 2019), however, the results are inconclusive. For some health outcomes such as allergic diseases, some studies reported negative impacts meaning higher associations between air pollution and health were observed in areas with higher levels of greenness (Cariñanos and Casares-Porcel 2011). Inconsistent findings may result from several factors such as different definitions of greenness and different metrics used to assess greenness across studies. Therefore, a comprehensive review of existing research on the impacts of greenness on the association between air pollution and health is needed to better understand the current state of the scientific literature on the complex relationship of greenness with air pollution and health outcomes.

In this study, we systematically reviewed previous literature on greenness as a potential effect modifier for associations between particulate matter air pollution and health outcomes. While previous literature reviews have focused on the direct impact of greenness on health outcomes such as mental health, mortality, and birth outcomes (Fong et al 2018, Zhan et al 2020), to the best of our knowledge, no previous systematic review synthesized the scientific evidence on the role of greenness as an effect modifier in the air pollution-health relationship. Such scientific evidence is important for policy makers to inform evidence-based interventions and policy to address the public health burden from air pollution and for management of greenspaces. Also, our study informs our understanding of gaps in knowledge and can identify research needs and directions for future study.

## 2. Methods

### 2.1. Search strategy

We conducted a systematic search using a MEDLINE/PubMed and Web of Science databases for population-based studies of greenspace as a modifier to the health impacts of particulate air pollution published until 30 November 2020. Our search protocol was as follows:

- ‘air pollution’, ‘air pollutant’, ‘air pollutants’, ‘PM_2.5_’, ‘PM_10_’, ‘fine partic*’, ‘particulate matter’, ‘airborne particles’ or ‘air quality’; AND
- mortality, death, ‘hospital admission’, ‘hospital admissions’, hospitalization, hospitalizations, birth, pregnancy, asthma, ‘birth weight’, ‘birth-weight’, ‘SGA’, ‘small for gestational age’, respiratory, cardiovascular, CVD, mental, cognitive, ‘lung function’, or stress; AND
- ‘green*’, vegetation, land, lands, ‘NDVI’, ‘EVI’ or ‘tree*’

where * indicates any combination of subsequent letters.

The search was conducted on 22 December 2020. The systematic search was conducted with consideration of the PRISMA (Preferred Reporting Items for Systematic Reviews and Meta-analyses) guidelines (Moher et al 2015).

### 2.2. Selection criteria

We selected studies meeting the following inclusion criteria. Studies had to: (a) be population-based; (b) consider exposure to particulate air pollution; (c) explore mortality or morbidity; (d) examine effect modification by greenness on the association between particulate matter air pollution and health; (e) be peer-reviewed; (f) be written in English; and (g) be published through November 2020. Both single-city and multicity studies were included.

**Figure 1.**
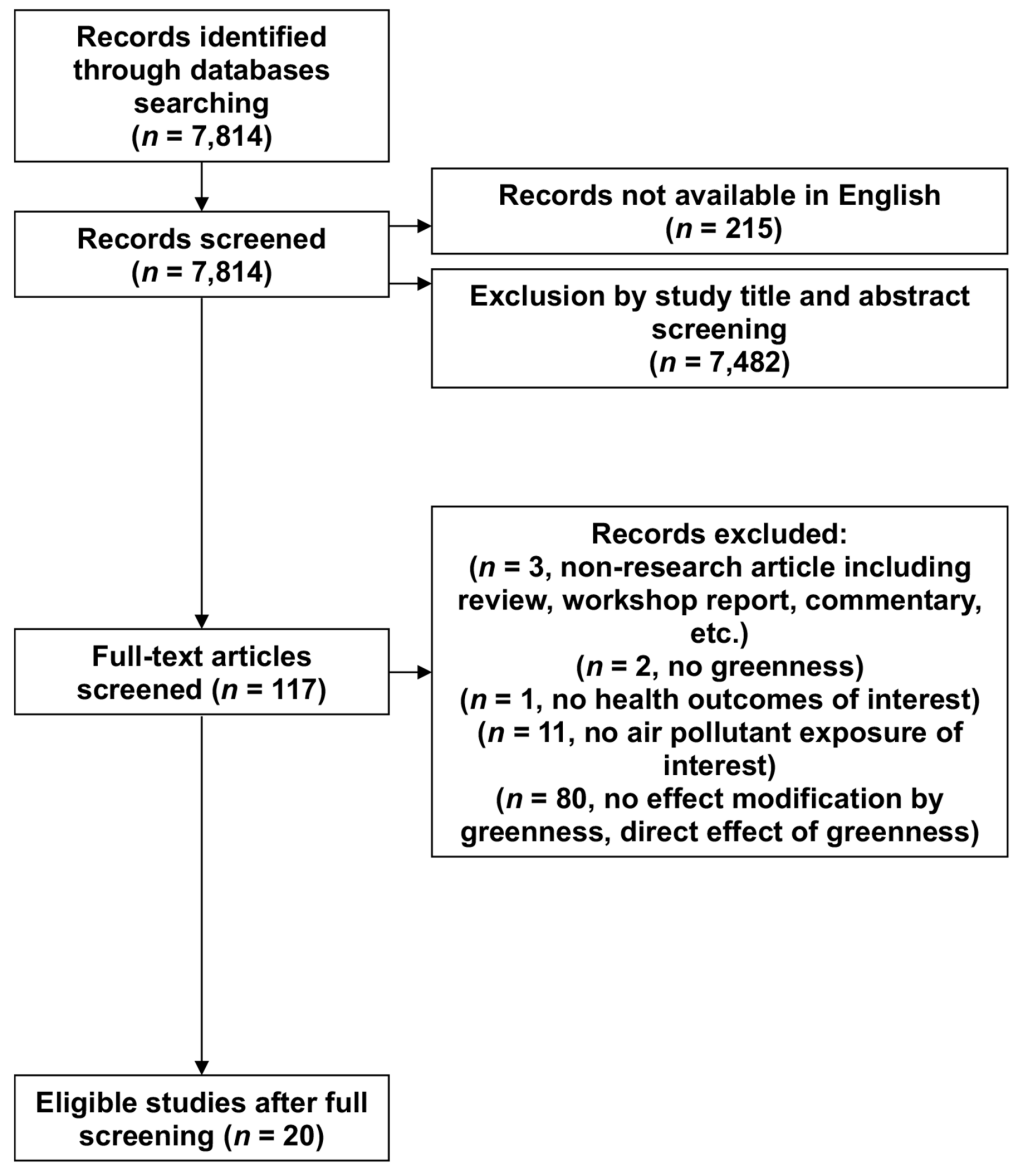
Flow diagram of literature selection process for systematic review. Note: Screening prior to ‘full-text’ screening was based on the title and abstract.

Screening of the articles was performed by two independent reviewers (CCL, HMC, JYS, KCF, and SH). Inconsistency regarding decisions of whether to include or exclude studies were resolved by a third reviewer (MLB). After we excluded studies by screening of titles and abstracts based on the inclusion criteria, we reviewed the full text of remaining articles. After the first initial screening (i.e. title and abstract), two reviewers independently performed full-text screening to determine whether the article was eligible for inclusion and, if so, performed data extraction. Figure 1 provides a flow diagram for the identification and selection of studies. After duplicate studies were removed, we initially identified and screened 7814 studies. Of these, 117 studies were identified for full-text screening, and of these 20 studies were eligible for inclusion in this review. We extracted information of each article’s study location, time frame, study design, health outcomes, air pollutant, air pollution exposure metric (e.g. daily average, annual concentration), increment of exposure used in presentation of effect estimates, lag structure (i.e. delayed effects of air pollution on health outcomes) (e.g. previous day, average of previous days), covariates adjusted in the model, effect modification factors studied, greenness metric (e.g. normalized difference vegetation index (NDVI), land use) and results for main findings regarding greenness as an effect modifier of the air pollution and health relationship. We used results from the key findings presented by study authors, as originally reported. Some studies could contribute more than one result, for example if they investigated multiple health outcomes.

We summarized findings on study characteristics based on several criteria: (a) health outcome: mortality, hospital admissions/emergency room visits, and other health outcomes; (b) air pollution exposure: PM_2.5_ (particulate matter with aerodynamic diameter ⩽2.5 *μ*m), PM_10_ (particulate matter with aerodynamic diameter ⩽10 *μ*m), and other; (c) source of exposure data: modeled, monitoring, and other; (d) study location; and (e) study time period. Study period was used to calculate the median year of the study period for each study. We evaluated characteristics of effect modification by greenness on the air pollution-health association based on (a) number of greenness metrics applied (i.e. single, multiple); (b) type of greenness metric (e.g. NDVI, land use); (c) source of data for greenness (e.g. moderate resolution imaging spectroradiometer (MODIS), Landsat); and (d) spatial resolution and buffer size (e.g. 250 m, county-level) for greenness estimate. For each study, we summarized evidence of effect modification of the particulate matter and health association by greenness based on the direction and strength of evidence based on the statistical significance and several study characteristics. Statistical significance was based on the main findings presented by study authors, as originally reported. If the authors did not state the statistical significance, we used numerical results (i.e. 95% confidence intervals, *p*-value of 0.05). We also summarized overall evidence of effect modification by greenness using the following categories: no evidence, weak evidence, limited/suggestive evidence, and strong evidence based on the quantity of studies providing consistent evidence compared with conflicting findings.

## 3. Results

A total of 7814 unique articles were identified from the systematic search (figure 1). After first screening these studies by title and abstract, 7482 papers were excluded. Non-English papers were also excluded (215 papers). After the initial screening (i.e. title and abstract), 117 papers remained for full-text review. The primary reasons for excluding studies at this stage were: no effect modification by greenness (e.g. study examined the direct effect of greenness), no air pollution exposure of interest, non-research article (e.g. review, commentary), no investigation of greenness, and no health outcomes of interest. The full-text screening resulted in 20 eligible studies for inclusion in our review.

### 3.1. Summary of study characteristics

Table 1 provides the characteristics of the studies included in this review: study period and location, study design, health outcome, air pollutant, air pollution exposure metric, lag structure, exposure increment used to present results, covariates adjusted in the model, potential effect modifiers considered, greenness metric, and main findings.

Table 2 shows summary characteristics across the 20 eligible studies for health outcomes, air pollutant, source of air pollution exposure data, study location, study period, and median year of the study period. The most represented country was the United States with six of the 20 studies. The remaining studies were conducted in China (four studies), the Netherlands (two studies), South Korea (two studies), Spain (two studies), and other countries (Canada, Greece, Iran, Italy). No study was conducted in South America or Africa. Most study time periods were less than five years (45%). Some studies examined multiple health outcomes and/or air pollution exposures. Among the 20 studies, 10 studies investigated mortality. Of the ten studies that examined mortality, seven considered non-accidental mortality. Two studies investigated hospital admissions/emergency room visits and eight studies assessed associations of air pollution with other health outcomes (e.g. preterm birth). For air pollution exposure, of the 20 studies, 16, 10, and 1 studies evaluated PM_2.5_, PM_10_, and other pollutants (e.g. coarse particles), respectively. Regarding the source of data for air pollution, 11 studies used modeled estimates and 10 studies used values from monitoring stations, with 1 study using estimates from both types of data sources.

Table 3 summarizes characteristics of the greenness metrics used in the studies based on whether a single or multiple greenness metric was applied, type of greenness metric, source of data for greenness, and spatial resolution and buffer size. Most studies (75%) applied a single greenness metric. NDVI was the most commonly used metric to quantify greenness (16 studies), followed by amount of green space/vegetation coverage based on land cover/land use data (4 studies). Other greenness metrics applied in the studies were distance between home and the boundary of the nearest green space, neighborhood walkability index, streetscape greenery, and landscape metrics. Nine studies used NDVI from MODIS, with spatial resolution of 250 m × 250 m, followed by NDVI from Landsat images with 30 m × 30 m spatial resolution (seven studies) and land use/land cover database/map (four studies). Some studies used distance from the nearest green space identified from a shapefile provided from the Municipality (one study), US EPA Smart Location database (one study), City Statistical Yearbook (one study), and street view image database (one study). Studies examined greenness at different spatial buffers. Eight studies applied multiple buffers, with others using various single buffer sizes (e.g. 250 m, 500 m, 1000 m) and administrative geographic areas (e.g. county level, census block group level, district).

### 3.2. Summary of evidence for effect modification by greenness on the association between air pollution and health

Table 4 provides a summary of evidence for effect modification by greenness on the association between particulate matter and health. Of the 20 studies, 8 studies (40%) found evidence of lower risk of air pollution on health outcomes in areas with high greenness. Three studies (15%) found higher risk of air pollution with high greenness and four studies (20%) did not find evidence of effect modification by greenness. Five studies (25%) showed different findings of the effect modification of air pollution and health associations by greenness depending on several factors such as air pollutants, urban/rural region, cause of death, and socioeconomic status (SES). For example, one study found higher risk of health impacts from PM_10_ and PM_2.5_ in areas with less greenness in rural regions, whereas estimated particulate matter health effects were higher in areas with more greenness in urban regions. Some studies found beneficial impact of greenness only in low SES areas.

We provided a summary of evidence for effect modification by greenness based on several study characteristics: health outcome, air pollutant, source of data for air pollution, study location, greenness metric (number and type), data source for greenness, and spatial resolution (table 5). Regarding health outcomes, we found suggestive evidence of lower risk with high greenness for the association between air pollution and mortality. Of the ten studies that investigated the risk of air pollution on mortality, five studies showed significant evidence of lower risk with high greenness, while one study reported significant evidence of higher risk with high greenness. For hospital admissions/emergency room visits or other health outcomes, there exists weak or no evidence of effect modification by greenness. Two studies examined the risk of air pollution on hospital admissions/emergency room visits, with one study showing significant evidence of lower risk with high greenness and one study reporting no evidence of effect modification. For air pollutants, we found suggestive evidence of lower risk with high greenness for both PM_2.5_ or PM_10_. Seven studies examining the effect of PM_2.5_ reported significant evidence of lower PM_2.5_-risk with high greenness or suggestive evidence of lower risk with high greenness, while one study reported significant evidence of higher risk with high greenness, four studies reported mixed evidence, and three studies identified no evidence of effect modification by greenness. We also observed suggestive evidence of effect modification by residential greenness (i.e. low risk with high greenness) in studies conducted in Asia or North America, using a single greenness metric, using NDVI as greenness metric, and using MODIS as data source for greenness.

## 4. Discussion

Our systematic review found that only a limited number of studies have investigated greenness as an effect modifier of the association between air pollution and health and that those studies included a range of study designs and characteristics such as the way in which greenness was assessed (e.g. greenness metrics applied, spatial resolution, and data sources). This review also observed suggestive evidence of the beneficial impacts of living in areas with high greenness with lower air pollution-health associations, although findings were inconsistent across studies.

### 4.1. Knowledge gaps in the evidence of effect modification by greenness and critical areas for future study

Although evidence on the direct effect of greenness and health outcomes is well documented (Banay et al 2017, Fong et al 2018, Zhan et al 2020, Hartley et al 2021), very few studies have been conducted to examine the effect modification by residential greenness on the association between air pollution and health outcomes. Findings of the studies we reviewed suggest that higher greenness was associated with lower risk of air pollution exposure for several health outcomes such as mortality, although results were not fully consistent. A recent study suggested an increasing trend in PM_10_ effects on birth outcomes with an increasing distance from green areas (Asta et al 2019). Another study in Canada evaluated the role of residential greenness in modifying associations between long-term exposure to PM_2.5_ and non-accidental and cause-specific mortality. They found that the strength of observed associations between PM_2.5_ and mortality decreased as greenness increased (Crouse et al 2019). Other studies also observed that risk of air pollution exposure on various health outcomes such as hospital admissions (Heo and Bell 2019) and infantile atopic dermatitis (Lee et al 2018) was lower for people living in areas with more green space.

Our findings of overall evidence of effect modification by greenness based on several study characteristics can inform understanding of knowledge gaps and help identify research needs and direction for future study. For example, we observed that the most examined health outcome was mortality, for which we found suggestive evidence of lower risk with higher greenness, and there were few studies investigating the modifying effect by greenness for other health outcomes such as hospital admissions/emergency room visits, for which we found weak evidence of effect modification. This indicates that further research is needed on morbidity outcomes. Our findings also showed that most studies were based on areas in Asia, North America, and Europe. Current epidemiologic evidence is very limited in many other regions, especially in South America or Africa, and studies are needed in these areas. The influence of greenness on the association between air pollution and health could differ by location due to several factors such as differences in pollution mixtures (e.g. PM_2.5_ chemical composition, co-pollutants), population characteristics, indoor/outdoor activity patterns, housing, and type of greenness. Variability in findings across studies may further relate to differences in population characteristics, air pollution composition, and vegetation, thus studies are needed in many regions and types of settings. Scientific evidence from this review has important implications for policy makers developing urban planning and health policy strategies, as we identified potential benefits from greenspace.

### 4.2. Assessment of greenness

In our assessment of greenness as an effect modifier on air pollution-health associations, we found differences in several characteristics such as greenness metrics, data source, and spatial resolution and buffer across studies. To assess greenness, most studies applied NDVI from MODIS (Kioumourtzoglou et al 2016, James et al 2017, MacNaughton et al 2017, Heo and Bell 2019, Kim et al 2019, Yitshak-Sade et al 2019) or Landsat (De Keijzer et al 2017, Vivanco-Hidalgo et al 2018, Asta et al 2019, Crouse et al 2019) images. Studies examining NDVI applied different spatial buffers (from 100 m to 3000 m) or assessed greenness at the administrative boundary (e.g. county-level, census block group level) but the associations did not vary by spatial resolution or buffer size based on sensitivity analyses within these studies and comparison across studies. Greenness was commonly measured for the area around the subject’s residential address (James et al 2017, Lee et al 2018, Vivanco-Hidalgo et al 2018, Asta et al 2019). Most studies used individual-level geocoded addresses or spatial units. A limited number of studies used land cover/land use databases to assess greenness such as amount of green space/vegetation coverage (Groenewegen et al 2018, Lee et al 2018). Other studies assessed distance between residence and the boundary of the nearest major green space, neighborhood walkability index, or green space per capita (Madaniyazi et al 2016, Asta et al 2019, Yitshak-Sade et al 2019). Some studies conducted analyses based on multiple greenness metrics (Asta et al 2019, Yitshak-Sade et al 2019). Importantly, none of the metrics represent the full dimension of greenness (e.g. vegetation type, access), and further may lead to imprecise assessment due to factors such as low spatial resolution, buffer size, and irregular greenness patterns (Laurent et al 2013). Further, greenness levels change over time. A recent study using different exposure windows for greenness metric (i.e. cumulative NDVI and contemporaneous NDVI) showed different associations for PM_2.5_ and mortality (Ji et al 2020). Thus, future study considering more detailed information such as greenness type, accessibility, and quality of local green space is needed to better understand how greenness may modify the air pollution-health association. While ‘quality’ of green space can be subjective, several studies have assessed features of green space that relate to quality including accessibility, maintenance, variation, colorfulness, pet areas, safety, absence of literature, walking paths, lighting, and other factors (Van Dillen et al 2012, Sugiyama et al 2016).

### 4.3. Heterogeneity in modifying effect of greenness by several characteristics

Findings were not consistent across studies. Some studies did not find evidence to support effect modification by greenness for the estimated effects of air pollution on health outcomes, with null or opposite findings of higher effect with higher greenness (Kioumourtzoglou et al 2016, Madaniyazi et al 2016, James et al 2017, MacNaughton et al 2017, Vivanco-Hidalgo et al 2018). Our findings suggested that greenness may modify the associations between air pollution and mortality differentially depending on the cause of death. Kim et al (2019) found that the effect of PM_10_ on cardiovascular mortality was stronger in districts with a lower level of greenness, whereas the effect of PM_10_ on non-accidental mortality was higher in districts with higher greenness. The authors suggested that possible explanations for these seemingly contradictory findings are: high greenness areas produce allergenic pollens, which subsequently interact with air pollutants; increased exposure to pesticides; transmission of infections by arthropod vectors; and excessive exposure to UV radiation, which could reduce the immune defenses (Lohmus and Balbus 2015, WHO 2016). Further studies are needed to understand the underlying mechanisms and factors that contribute to these variations, with attention to the multiple pathways through which greenness could modify the air pollution and health relationship.

In our review, we found differences in the estimated modifying effect by greenness between urban and rural areas. For example, de Keijzer et al (2017) found that effects of PM_10_ and PM_2.5_ were higher in areas with less greenness in rural regions, whereas those effects were higher in areas with more greenness in urban regions. The different patterns between the urban and rural areas may result from differences in population characteristics (e.g. indoor/outdoor activity patterns, health behaviors), PM_2.5_ chemical composition relating to different sources, type and diversity of greenness, and other environmental factors that differ between urban and rural areas.

Previous literature suggested that health benefits related to greenness may differ by socioeconomic status. Yitshak-Sade et al (2019) observed that PM_2.5_-related CVD mortality risk was lower in highly populated greener neighborhoods with sociodemographic features that are highly correlated with lower socioeconomic status. Another study reported that in low SES areas, green space seems to alleviate effects of air pollution on the prevalence of high blood pressure and diabetes (Groenewegen et al 2018). Intersectionality between SES and other population characteristics relating to vulnerability (e.g. race/ethnicity, rurality) may contribute to the health disparities through multiple pathways. Thus, further research is needed considering relevant factors and complex interaction in relation with underlying mechanisms.

### 4.4. Possible mechanisms of modifying effect by greenness

There are several possible mechanisms through which greenness could modify the air pollution and health relationship, such as changes to the air pollution mixture (e.g. filtering of pollutants), different exposure patterns, and increased overall health (e.g. physical activity, mental health and wellbeing) in ways that affect susceptibility. Greenness may reduce the adverse effect of air pollution by filtering of air pollutants and increased dispersal of pollutants through improving ventilation, improving the immune system through psychological restoration for stress and anxiety, and increasing physical activity and social cohesion (Nowak et al 2006, Nieuwenhuijsen et al 2014, Markevych et al 2017). Another possible mechanism is the environmental biodiversity hypothesis. This suggests that contact with nature is beneficial for human microbiota, which influence immune tolerance and immunomodulatory capacity (Rook 2013, Kuo 2015). A study suggested that increasing greenness and lowering air pollution might have a synergistic effect on mortality and interact non-linearly with mortality (Ji et al 2020). Other studies reported mixed findings on whether greenness mediates air pollution-related exposure pathways, which indicate the complex relationships between greenness, air pollution, and health outcomes (de Keijzer et al 2017, Kim et al 2019). Further studies are needed to understand the underlying physiological mechanisms of how greenness could modify the association between air pollution and health.

### 4.5. Study limitations

Our review includes several limitations. We were unable to quantitatively combine estimates through meta-analysis due to the small number of studies with the same category of health outcome, particulate matter exposure, and assessment of greenness. We elected not to assess quality of the identified studies. Instead, we provided detailed information for each individual study such as health outcome, air pollution exposure, exposure data source, greenness metric and data source, and spatial resolution and buffer size for greenness measurement. This information from our review provides valuable knowledge on how greenness affects associations between air pollution and health outcomes and helps identify critical areas for future study. Publication bias may exist. Studies that did not find statistically significant results or found unanticipated findings may be selectively less likely to be submitted or published. To the best of our knowledge, this is the first systematic review of effect modification by greenness on the association between air pollution and health outcomes. The findings from our review extend our understanding of greenness as an effect modifier, provide vital information for policy makers, and inform future studies.

## 5. Conclusion

Overall, our review suggests that greenness may have positive effects on the association between air pollution and health outcomes, although some findings vary, such as by type of health outcome, and results were inconsistent. Future research considering multiple relevant factors and pathways is needed to better understand the characteristics, mechanisms, and potential mediators in relation to greenness on the association of air pollution and health outcomes.

## Figures and Tables

**Table 1.** Studies included in the systematic review.

Study (reference no)	Study location and period	Study design	Health outcomes	Air pollutant	Air pollution exposure metric and lag	Air pollution increment for comparison	Covariates adjusted	Effect modifiers	Greenness metric	Main findings
Asta et al (2019) [1]	Rome, Italy. April to October 2001–2013	Cohort	Preterm births between the 22nd and 36th week of gestation	PM_10_	Daily values of PM_10_ (24 h mean). Lag 12–22 days for PM_10_	1 *μ*g m^−3^ PM_10_	Long term trend, seasonality, holidays, daily maximum apparent temperature	Socioeconomic position, greenness	NDVI within 100 m buffer around mother’s residential address: minimum straight line distance between home addresses and boundary of nearest major green space, classified as within 100, 100–500, and >500 m from green spaces; Landsat 8-OLI/TIRS remote sensing image of NDVI from USGS database, with 30 × 30 m spatial resolution, summer day, calculated average NDVI in each 100 m buffer around mothers’ residential addresses and classified using NDVI’s tertiles	None of the considered green space factors were associated with modified estimated effect of PM_10_ on preterm birth. However, results suggested an increasing trend in PM_10_ effects with increasing distance from green areas.
Crouse et al (2019) [2]	Canada. 2001–2011	Cohort	All non-accidental causes (ICD-10: A to R); cardiometabolic (i.e. circulatory plus diabetes; ICD-10: I10 to I69, E10 to E14); and CVD diseases (ICD-10: I10 to I69) mortality	Satellite-derived annual estimates of PM_2.5_ gridded at ~1 × 1 km spatial resolution to individuals’ six-digit residential ZIP codes	Three-year moving averages with one-year lag (and updated annually for residential mobility)	IQR (3.5 *μ*g m^−3^)	Visible minority status, aboriginal status, marital status, education, income quintile, employment status, neighourhood deprivation, community size, airshed	Sex, five-year age group, greenness	NDVI within 500 m, across quintiles of greenness, models stratified by high and low community deprivation and greenness	Level of observed PM_2.5^−^_ mortality association decreased as greenness increased. This pattern persisted in models restricted to urban residents and within neighborhoods characterized by high or low deprivation. Increased mortality risk associated with PM_2.5_ was not observed among those living in the greenest areas. For example, HR for CVD mortality among individuals in the least green areas was 1.17 (95% CI: 1.12–1.23) compared to 1.01 (95% CI: 0.97–1.06) in the greenest areas.
De Keijzer et al (2017) [3]	Spain. 2009–2013 for mortality, 2010–2012 for life expectancy	Ecological	Standardized mortality rate and life expectancy, natural causes (ICD-9: 001–799, ICD-10: A00–R99)	PM_10_, PM_2.5_ obtained from CALIOPE air quality forecasting system (1 h and 4 × 4 km resolution)	Annual PM_10_, PM_2.5_	5 *μ*g m^−3^ for PM_10_; 2 *μ*g m^−3^ for PM_2.5_	Socioeconomic vulnerability index, percentage of people with low education, lung cancer SMRs as a proxy for smoking	Greenness	NDVI from Landsat8 at 30 × 30 m resolution	Effects of PM_10_ and PM_2.5_ were higher in areas with less greenness in rural regions, whereas those effects were higher in areas with more greenness in urban regions. Protective associations of greenness with mortality and longer life expectancy was only found in areas with lower socioeconomic status.
Groenewegen et al (2018) [4]	Netherlands. 2013	Cross-sectional	Ten clusters of health problems: cardiovascular morbidity (high blood pressure (K85, 86, 87), cardiac disease (K71, 73, 74, 77–84), coronary heart disease (K74-76), stroke and brain hemorrhage (K89, 90)), mental health problems (depression (P03, 76), anxiety disorder (P01, 74)), respiratory disorders (asthma, COPD (R91, 95, 96)), neurological disorders (migraine/several headache (N01-03, 89, 90, 92)), medically unexplained physical symptoms (A01, A04, D01, D08, D09, D12, D18, D21, D93, K01, K02, K04, L01-03, L08, L09, L14, L20, N01, N02, N17, P06, P20, R02, R21, T03, T07, T08), diabetes (T88, T90)	PM_10_ and PM_2.5_ at home address estimated by land use regression models (ESCAPE study)	95th percentile of the distribution of modelled PM_10_, PM_2.5_, NO_2_ levels	1 *μ*g m^−3^ for PM_10_ and PM_2.5_	Age, sex, area-level confounders (area SES, percentage immigrants, population density, urbanicity of municipality)	Green space, unsafety feeling, area-level SES	Green space from national land use database; total green space (agricultural green, woods and nature areas) as % of total number of grid cells (25 m × 25 m) in each ZIP code area	In low SES areas, green space appeared to alleviate effects of air pollution on prevalence of high blood pressure and diabetes.
Heo and Bell (2019) [5]	364 urban US counties. 2000–2013	Time-series	Hospital admissions for CVD or respiratory diseases from Medicare enrollees (⩾65 y), all CVD diseases (ICD-9 390–459), acute myocardial infarction (AMI) (410), ischemic heart disease (410–414, 429), heart failure (428), and stroke (430–438), all respiratory diseases (460–519), and respiratory tract infections (464–466, 480–487)	Daily PM_2.5_ and PM_10_ at the county level	Daily values. Lag 0	10 *μ*g m^−3^	County-specific model: day of the week, time, daily mean temperature, daily mean dew point temperature, offset term of Medicare population at risk; meta-regression: median household income, percent of the population ⩾65 years, percent of persons ⩾65 years in poverty, offset term for population density, latitude of the county	Sex, age group, greenness	NDVI at 250 m × 250 m resolution, county-specific values	Association between air pollution and health was lower in areas with more green space. IRQ increase in NDVI corresponds to 1.68% (95% CI: 0.43, 2.91) decrease in association between PM_10_ and CVD hospitalization and 10.40% (95%CI: 7.34,13.34) decrease in PM_10^−^_ hospitalization association of AMI. For hospitalization associated with PM_2.5_, a 0.18% (95%CI:–0.39,0.73) absolute decrease in relative risk was found for CVD hospitalizations. Study found evidence for health benefits of green space for diminishing health impacts of particulate matter on hospitalizations of older persons.
Jaafari et al (2020) [6]	Tehran, Iran 2017	Cross-sectional	Respiratory disease mortality and respiratory cancer mortality	Hourly measurements for PM_10_ and PM_2.5_ from 39 air quality monitoring stations; ordinary kriging interpolation method used	Five-year averages	N/A	N/A	Structural equation modeling approach—significant mediation	Five landscape metrics (total class area, cohesion index, patch density, shape index, total edge) for urban green space from land use/cover map	Green space had a direct negative effect on air pollution (green space → air pollution (−0.836)). Green space has a significant mitigating effect on air pollution and mortality of respiratory diseases.
James et al (2017) [7]	US, mostly New England. 2006	Cohort	BMI, self-reported	Modeled PM_2.5_	2006 annual average at each participant’s residence	N/A	Age, race, smoking status, individual-level SES (husband’s education), area-level SES (median household income, home value, proportion of residents without a high school diploma)	Assessed potential interactions between pairs of exposures (air pollution with greenness)	NDVI from MODIS—one image every 16 days at 250 m resolution—Summertime July 2006 image was used; based on participant’s geocoded address and corresponding NDVI image pixel	Authors did not find evidence to support effect modification by greenness for the estimated effects of PM_2.5_ on BMI.
Ji et al (2020) [8]	631 cities and counties, China. 2008–2014	Cohort	All-cause mortality	Modeled PM_2.5_ at 1 × 1 km spatial resolution	Three-year average before death or the end of follow-up	10 *μ*g m^−3^	Age, sex, ethnicity, marital status, geographical region, urban or rural residence, education, main occupation before 60 years of age, financial support, social and leisure activity, smoking status, alcohol consumption, physical activity	Greenness	NDVI from MODIS—500 m radius around each participant’s residence—calculated two NDVI values every month for each season and computed contemporaneous NDVI which is the value at the time closest to an event	There was evidence for a synergistic effect from greenness and air pollution. In analyses stratified by NDVI tertiles, the estimated effect of PM_2.5_ on mortality was lowest in the highest NDVI tertile, especially when restricted to urban areas only.
Kasdagli et al (2021) [9]	Greece 2011	Ecological	All natural-causes excluding deaths from external (ICD-9: 001-799, ICD-10: A00-R00); cardiovascular (ICD09: 390-459, ICD-10: I00-I99); respiratory (ICD-9: 460-519, ICD-10: J00-J99) mortality	PM_2.5_ were derived from 100 × 100 m surfaces predicted by hybrid LUR models	Annual average for 2010	IQR (3.34 *μ*g m^−3^)	Lung cancer rates, population born in Greece, unemployment rate, population aged 25–64 years with upper secondary or tertiary education attainment, urbanicity	Greenness	NDVI from NASA MODIS at 1 km spatial resolution and 16 days temporal resolution; average NDVI for each municipal unit level	Negative interaction between NDVI and PM_2.5_ for cardiovascular mortality; areas with higher greenness were associated with lower PM_2.5_ effects on cardiovascular mortality.
Kim et al (2019) [10]	Seven metropolitan cities (Seoul, Busan, Daegu, Incheon, Gwangju, Daejeon, and Ulsan), South Korea. 2008–2016	Time-series	Non-accidental (ICD10; A00-R99), CVD disease (I00-99), ischemic heart disease (I20-25), respiratory disease (J00-99), chronic lower respiratory disease (J40-47), and lung cancer (C33-34) mortality	PM_10_	Annual mean concentration of PM_10_ from daily 24 h mean concentrations for each district	10 *μ*g m^−3^ and IQR	Neighborhood SES, smoking rate, healthcare infrastructure status	Greenness	Median value NDVI for summer (May–October), which indicates greenness level in peak bloom, greenness was categorized into tertiles to obtain sufficient data within each category and practical interpretation (low/medium/high) of values	Associations between PM_10_ and mortality varied by level of greenness. However, greenness modified associations differently depending on cause of death. PM_10_ non-accidental mortality association was higher in districts with higher greenness. However, estimated effect of PM_10_ on CVD mortality was higher in districts with lower greenness, although interaction terms were not significant.
Kioumourtzoglou et al (2016) [11]	207 US cities. 2000–2010	Cohort	Total mortality, Medicare data (⩾65 y)	PM_2.5_ from monitoring sites	Annual and two-year average	10 *μ*g m^−3^	Calendar year, previous admission for CHF, COPD, MI or diabetes, number of days spent in the intensive and coronary care units, ZIP code level median income	Temperature and city-level variables, including smoking and obesity rates, poverty, education, urbanicity and greenness	Mean vegetation index (NDVI) by county for representative month in each season (January, April, July, and October) over study period: two vegetation index metrics as averages over 2000–2010: annual and summer average vegetation index	Positive modification of greenness on PM_2.5_-mortality associations; greenness was positively correlated with proportion of black and elderly residents and smoking rates. These could contribute to the observed positive modification. In an analysis within regions, however, greenness was not a positive modifier in any region and was related to lower effect estimates in the Northeast and the Southwest.
Klompmaker et al (2019) [12]	Netherlands 2012	Cross-sectional	Self-reported cardiometabolic diseases (diabetes, hypertension, stroke, and heart attack)	PM_10_, PM_2.5_, and PM_coarse_, PM_2.5abs_ for each home address estimated by land use regression models developed within the framework of the ESCAPE project, two oxidative potential (OP) metrics: electron spin resonance (OP^ESR^) and dithiothreitol (OP^DTT^)	Long-term average in 2009, three years prior to the health survey—no LUR models available for 2012	IQR (PM_2.5_ 0.83 *μ*g m^−3^; OP^DTT^ 0.27 nmol DTT min m^−3^)	Sex, age, marital status, region of origin, education, work, standardized household income, smoking habits, number of cigarettes/day, alcohol consumption, number of alcohol glasses/week, physical activity, BMI, neighborhood SES	Greenness, traffic noise	NDVI derived from LANDSAT 5 TM (30 m × 30 m resolution) from the summer of 2010; proportion of green space from national land-use database—assessed buffers with radii of 100, 300, 500, 1000, and 3000 m	The ORs of diabetes prevalence per 0.27 increase in OP^DTT^: Q1 (lowest NDVI) 1.00 (0.94, 1.05), Q2 1.01 (0.93, 1.09), Q3 1.08 (1.00, 1.17), Q4 1.08 (1.00, 1.17), Q5 (highest NDVI) 1.09 (1.01, 1.18); higher effects of OP^DTT^ were found in the higher quintile of NDVI.
Lee et al (2018) [13]	South Korea. 2006–2010	Cohort	Infantile atopic dermatitis, ICD code not provided	PM_10_ derived from land use regression model	Mean PM_10_; first (0–12 weeks gestation), second (13–28), and third (29–42) trimester; and entire pregnancy (0–42 weeks)	10 *μ*g m^−3^	Maternal age, education, income, body mass index, history of allergy, exposure to secondhand smoke, residential mobility, gestational age, the presence of pets, infant sex, birth weight, season of birth, breastfeeding, mode of delivery, temperature, humidity	Green space	Amount of green space within buffers from residential home (100, 200, 300, 500 m) based on GIS land cover data	Stratified analysis revealed that risk of atopy associated with PM_10_ exposure during 1st trimester decreased with increase in green space in residential areas.
MacNaughton et al (2017) [14]	Massachusetts, US. 2012–2013	Cross-sectional	Chronic absenteeism (reported from elementary and secondary public schools)	PM_2.5_ (modeled from satellite-based data)	Average PM_2.5_ concentration, 1 September, 2012 to 30 June, 2013 at 1 km resolution	IQR (1 *μ*g m^−3^)	Race, income	Greenness	NDVI at 250 m × 250 m resolution from NASA’s MODIS	Interaction term showed higher effect of PM_2.5_ when NDVI is high and dampening of PM_2.5_ effect when NDVI is low.
Madaniyazi et al (2016) [15]	73 cities, China. 2010	Cross-sectional	Total, respiratory, and CVD mortality	PM_10_ from monitoring stations	Mean two-day moving average	10 *μ*g m^−3^	City-specific characteristics	Annual green space per capita and gross domestic product (GDP) per capita	Green space per capita (public green space + productive plantation area + green buffer space + attached green space)/population	Green space per capita could explain the heterogeneity in PM_10^−^_mortality associations. Coefficients of green space per capita were positive and statistically significant, suggesting that people living in cities with more green space per capita have higher PM_10_-respiratory and CVD mortality associations.
Sun et al (2020a) [16]	Hong Kong 1998–2011	Cohort	Respiratory (J00-J47, J80-J99), pneumonia (J12-J18), and chronic obstructive pulmonary diseases (COPD, J40-J44, J47) mortality	PM_2.5_ and PM_10_ obtained from 10 general air monitoring stations	Daily mean concentration. Four-day moving averages (lag 0–3)	10 *μ*g m^−3^	Long-term trend and seasonality (control period as the same year, month, day of the week as the case); temperature, relative humidity, influenza epidemics, public holidays	Greenness	NDVI derived from Landsat 5 TM (30 m × 30 m resolution) at 2001 and 2006—mean NDVI within a buffer of 250 m and 500 m radius around residential address	The mortality risk of pneumonia showed a decreasing trend for PM_2.5_ (p for trend = 0.034) with greenness quartiles increasing from Quartile 1 (lowest) to Quartile 4 (highest); elders living in higher greenness areas are less susceptible to pneumonia mortality associated with air pollution.
Sun et al (2020b) [17]	California, US 2001–2008	Cohort	Preterm birth (<37 weeks)	Hourly PM_2.5_ from US EPA’s monitoring stations	Daily average (24 h) and then monthly average was calculated; monthly average concentrations of PM_2.5_ was spatially interpolated between stations using an empirical Bayesian kriging (EBK) model.	10 *μ*g m^−3^	Maternal age, race/ethnicity, educational level, median household income, maternal address ZIP code	Green space	MODIS NDVI data—spatial resolution of 250 m × 250 m and temporal resolution of every eight-day—average NDVI calculated based on circular buffers of 250, 500, 1000, 2000 m of each residential location	Consistent positive multiplicative and additive interactions were observed between decreasing green space and higher air pollution.
Vivanco-Hidalgo et al (2018) [18]	Barcelona, Spain. 2005–2014	Case-crossover	Ischemic stroke and subtypes in a cohort of hospital admissions from ischemic stroke or transient ischemic attacks, ICD code not provided	PM_2.5_ from monitoring site	Hourly PM_2.5_. Up to previous 72 h before stroke symptom onset (Lag 0, 12, 24–47, and 48–72 h)	IQR (0.9–2.4 *μ*g m^−3^)	Apparent temperature	Green space, noise	Average NDVI within 100, 300, and 500 m of participan’s address	No association observed between PM_2.5_ exposure and acute ischemic stroke risk. No clear modifying effect of greenness or noise was observed.
Wang et al (2020) [19]	Guangzhou, China Enrolled study participants between June and August 2016; PM_2.5_ and NDVI data in 2016	Cross-sectional	Psychological well-being using survey data (WHO 5 items Well-Being Index questions)	Predicted PM_2.5_ concentration within a 1000 m circular buffer around the geographic centroid of study neighborhoods using MODIS AOD data	Annual average	N/A	Sex, age, educational attainment, marital status, hukou status, annual household income, medical insurance participation	Greenness	NDVI from Landsat 8 OLI and TIRS at 30 m × 30 m resolution, calculate the NDVI in 1000 m buffers around the centroid of each study neighborhood; streetscape greenery (SVG)—trees (SVG-tree) and grasses (SVG-grass) from street view image database (Tencent Online Map)	There was no evidence to suggest that NDVI could influence WHO-5 scores through an indirect effect.
Yitshak-Sade et al (2019) [20]	Massachusetts, US. 2001–2011	Case-crossover	CVD mortality (ICD-10 group I), ⩾40 y	PM_2.5_ from model at 1 × 1 km spatial resolution	Average daily PM_2.5_. Lag 0–1	Each 10 *μ*g m^−3^ increase in PM_2.5_ at the 25th and 75th percentiles of the land-use modifier within each stratum (above/below median value) of neighborhood sociodemographic characteristic	Day of the week, temperature	Neighborhood land-use characteristics and sociodemographic characteristics (% of residents by race (white, black or other), % of residents with no high school diploma, median household income, % of residents below poverty line, and population density)	Annual average of NDVI (250 × 250 m resolution), 250 m and 1250 m buffer; Neighborhood walkability index at Census block group-level	Statistically significant association between PM_2.5_ and CVD mortality. Among all cases, risk was not modified by neighborhood greenness or walkability. However, PM_2.5_-related CVD mortality was lower with higher NDVI only in neighborhoods with higher population density, lower % of white population, or higher % of residents without a high school diploma. Although interaction between greenness and PM_2.5_-mortality risk was not statistically significant, authors observed smaller PM_2.5_ effects in greener neighborhoods with higher % of poverty and lower median household income. No joint effect modification was observed for walkability.

*Notes:* Abbreviations: AMI (acute myocardial infarction), BC (black carbon), BMI (body mass index), CVD (cardiovascular), HR (hazard ratio), ICD (International Classification of Diseases Code), IQR (interquartile range), lag (lag 0 represents exposure the same day as health event, lag 1 the previous day’s exposure, etc.), PM_10_ (particulate matter with aerodynamic diameter ⩽10 *μ*m), PM_2.5_ (particulate matter with aerodynamic diameter ⩽2.5 *μ*m), SES (socio-economic status), USGS (United States Geological Survey). Study numbers in column 1 are used to refer to specific studies in subsequent tables.

**Table 2.** Summary of study characteristics across 20 studies included in this review.

Criterion	Number of studies (%)	Study reference numbers^f^
**Health outcomes**		
Mortality	10 (50%)	2, 3, 6, 8, 9, 10, 11, 15, 16, 20
Non-accidental	7	2, 3, 8, 9, 10, 11, 15
Cardiovascular	5	2, 9, 10, 15, 20
Respiratory	5	6, 9, 10, 15, 16
Other mortality^a^	5	2, 3, 6, 10, 16
Hospital admissions/emergency room visits	2 (10%)	5, 18
Cardiovascular	1	5
Respiratory	1	5
Other^b^	1	18
Other health outcome^c^	8 (40%)	1, 4, 7, 12, 13, 14, 17, 19
**Air pollutant** ^g^		
PM_2.5_	16 (80%)	2, 3, 4, 5, 6, 7, 8, 9, 11, 12, 14, 16, 17, 18, 19, 20
PM_10_	10 (50%)	1, 3, 4, 5, 6, 10, 12, 13, 15, 16
Other^d^	1 (5%)	12
Air pollutant exposure data source^g^		
Modeled	11 (55%)	2, 3, 4, 7, 8, 9, 12, 13, 14, 19, 20
Monitors	10 (50%)	1, 5, 6, 7, 10, 11, 15, 16, 17, 18
Other^e^	1 (5%)	12
**Study location**		
Canada	1 (5%)	2
China	4 (20%)	8, 15, 16, 19
Greece	1 (5%)	9
Iran	1 (5%)	6
Italy	1 (5%)	1
Netherland	2 (10%)	4, 12
South Korea	2 (10%)	10, 13
Spain	2 (10%)	3, 18
USA	6 (30%)	5, 7, 11, 14, 17, 20
**Study period**		
<5 years	9 (45%)	3, 4, 6, 7, 9, 12, 14, 15, 19
5–9	4 (20%)	8, 10, 13, 17
10–14	7 (35%)	1, 2, 5, 11, 16, 18, 20
**Median year of study period**		
2004–2007	8 (40%)	1, 2, 5, 7, 11, 16, 17, 20
2008–2011	6 (30%)	3, 8, 9, 13, 15, 18
⩾2012	6 (30%)	4, 6, 10, 12, 14, 19

a Cardiorespiratory (ICD-10 I00-I99 and J00-J99), mental and behavioral disorders (F00-F99), nervous system (G00-G99), skin and subcutaneous tissue (L00-L99), life expectancy, ischemic heart disease (I20-I25), chronic lower respiratory disease (J40-J47), lung cancer (C33-C34), cardiometabolic (I10-I69, E10-E14) cause, respiratory cancer mortality, respiratory (J00-J47, J80-J99), pneumonia (J12-J18), and chronic obstructive pulmonary diseases (COPD, J40-J44, J47) mortality.

b Acute ischemic stroke and subtypes (large-artery atherosclerosis, small-vessel occlusion, cardioembolism, other determined cause, or undetermined cause).

c Atopic dermatitis by the questionnaire, chronic absenteeism, general practitioner-assessed cardiovascular morbidity, respiratory disorders, mental health problems, diabetes, and other physical symptoms, preterm birth, term birth weight, BMI, affirmative asthma identified by survey, self-reported cardiometabolic diseases (diabetes, hypertension, stroke, and heart attack), psychological well-being using survey data.

d PM_coarse_, PM_abs_, OP^ESR^, OP^DTT^.

e Oxidative potential (OP).

f Study reference numbers refer to specific studies as noted in the first column of table 1. Some studies contributed more than one results and are represented in multiple rows.

g Not all categories are mutually exclusive; thus, the sum of percentages may exceed 100%.

**Table 3.** Summary of greenness metric.

	Number of studies (%)	Study reference numbers^c^
**Greenness metric**		
Applied a single metric	15 (75%)	2, 3, 4, 5, 7, 8, 9, 10, 11, 13, 14, 15, 16, 17, 18
Applied multiple metrics	5 (25%)	1, 6, 12, 19, 20
**Greenness metric type**		
NDVI	16	1, 2, 3, 5, 7, 8, 9, 10, 11, 12, 14, 16, 17, 18, 19, 20
Amount of green space/vegetation coverage	4	4, 12, 13, 15
Distance between home and the boundary of the nearest major green space	1	1
Neighborhood walkability index	1	20
Streetscape greenery	1	19
Landscape metrics	1	6
**Data source for greenness**		
MODIS	9	5, 7, 8, 9, 10, 11, 14, 17, 20
Landsat 5 or 8	7	1, 2, 3, 12, 16, 18, 19
Land cover/land use database/map	3	4, 6, 13
Other^a^	4	1, 15, 19, 20
**Spatial resolution and buffer**		
250 m surrounding each residence/school	1	14
500 m	2	2, 8
1000 m around the centroid of each neighborhood	1	19
Multiple buffers (from 100 m to 3000 m)	8	1, 7, 12, 13, 16, 17, 18, 20
Census block group level	1	20
County-level	2	5, 11
Dominant type of land use of each 25 m × 25 m grid cell	1	4
Other^b^	5	3, 6, 9, 10, 15

a Distance from the nearest major green space identified from a shapefile provided from Municipality of Rome, neighborhood walkability index combining *z*-scores of three components (population density on unprotected land, street intersection density, and land-use diversity) from the US EPA Smart Location Database, China City Statistical Yearbooks, street view image database (Tencent Online Map), streetscape greenery data gathered via field surveys.

b Teritary Planning Unit (TPU) with percentage of vegetation < or ⩾ 50th percentile, percentage of land-use within the SA2 (Statistical Area 2) developed by the Australian Bureau of Statistics (SA2s are a surrogate spatial representation for local communities, with ~10 000 residents on average), small geographical areas (municipalities or if <3500 inhabitants, groups of adjacent municipalities with similar social and demographic characteristics, large cities included as single areas), district (gu/gun) (an administrative unit in Korea similar to borough in European and US cities) then categorized into tertiles, Chinese cities, the cell size of green area layers was 10 m, average NDVI for each municipal unit level, green structure category in 48 districts (largest patch percentage, landscape proportion, aggregation, patch distance, fragmentation).

c Study reference numbers refer to specific studies as noted in the first column of table 1. Some studies contributed more than one results and are represented in multiple rows.

**Table 4.** Findings of effect modification by greenness.

Summary of evidence	Number of studies (%)	Study reference numbers^b^
Evidence of lower risk with high greenness	8 (40%)	2, 5, 6, 8, 9, 13, 16, 17
Evidence of higher risk with high greenness	3 (15%)	12, 14, 15
No evidence of effect modification	4 (20%)	1, 7, 18, 19
Different findings by factors or only found in specific group^a^	5 (25%)	3, 4, 10, 11, 20

a Different findings by several factors such as air pollutants, urban/rural, region, or cause of death, or only found effect modification by greenness in specific group (e.g. higher risk in areas with less greenness in rural regions, whereas higher risk in areas with more greenness in urban regions, beneficial impact only in low SES areas).

b Study reference numbers refer to specific studies as noted in the first column of table 1.

**Table 5.** Summary of evidence for effect modification by greenness on the association between particulate matter and health, by study characteristics.

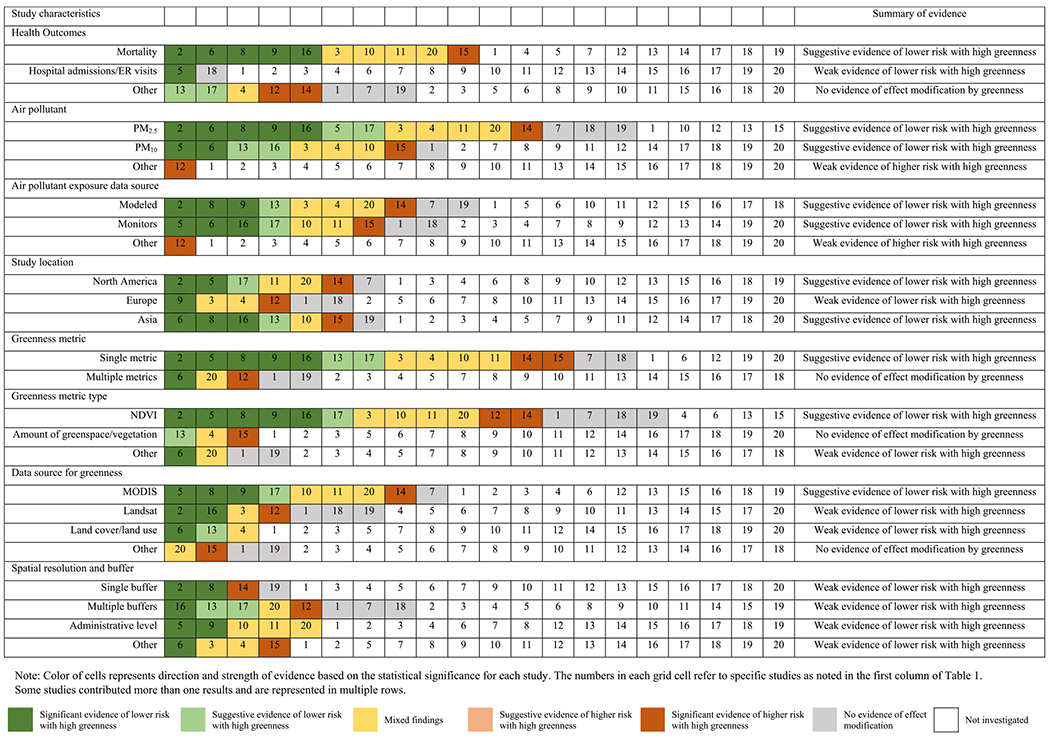

Note: If the study reported mixed findings, we included information on each case separately but for some common study characteristics, we used the color of stronger evidence; We summarized overall summary of evidence using the following categories: no evidence, weak evidence, limited/suggestive evidence, and strong evidence based on the quantity of studies providing consistent evidence compared with conflicting findings.

## Data Availability

No new data were created or analyzed in this study.
